# The Danish Future Patient Telerehabilitation Program for Patients With Atrial Fibrillation: Design and Pilot Study in Collaboration With Patients and Their Spouses

**DOI:** 10.2196/27321

**Published:** 2021-07-19

**Authors:** Birthe Dinesen, Josefine Dam Gade, Cathrine Skov Schacksen, Helle Spindler, Andi Eie Albertsen, Lars Dittmann, Mads Jochumsen, Dorthe Svenstrup Møller

**Affiliations:** 1 Department of Health Science and Technology Laboratory for Welfare Technology - Telehealth & Telerehabilitation, Sport Sciences - Performance and Technology Aalborg University Aalborg East Denmark Denmark; 2 Department of Psychology and Behavioral Sciences Aarhus University Aarhus Denmark; 3 Department of Cardiology Viborg and Skive Regional Hospital Viborg Denmark; 4 Department of Photonics Engineering Danish Technical University Copenhagen Denmark

**Keywords:** atrial fibrillation, cardiac rehabilitation, telerehabilitation, patient education

## Abstract

**Background:**

Atrial fibrillation (AF) is the most common cardiac arrhythmia and is predicted to more than double in prevalence over the next 20 years. Tailored patient education is recommended as an important aspect of AF care. Current guidelines emphasize that patients become more active participants in the management of their own disease, yet there are no rehabilitation programs for patients with AF in the Danish health care system. Through participatory design, we developed the Future Patient Telerehabilitation (TR) Programs, A and B, for patients with AF. The 2 programs are based on HeartPortal and remote monitoring, together with educational modules.

**Objective:**

The aim of this pilot study is to evaluate and compare the feasibility of the 2 programs of TR for patients with AF.

**Methods:**

This pilot study was conducted between December 2019 and March 2020. The pilot study consisted of testing the 2 TR programs, A and B, in two phases: (1) treatment at the AF clinic and (2) TR at home. The primary outcome of the study was the usability of technologies for self-monitoring and the context of the TR programs as seen from patients’ perspectives. Secondary outcomes were the development of patients’ knowledge of AF, development of clinical data, and understanding the expectations and experiences of patients and spouses. Data were collected through interviews, questionnaires, and clinical measurements from home monitoring devices. Statistical analyses were performed using the IBM SPSS Statistics version 26. Qualitative data were analyzed using NVivo 12.0.

**Results:**

Through interviews, patients articulated the following themes about participating in a TR program: usefulness of the HeartPortal, feeling more secure living with AF, community of practice living with AF, and measuring heart rhythm makes good sense. Through interviews, the spouses of patients with AF expressed that they had gained increased knowledge about AF and how to support their spouses living with AF in everyday life. Results from the responses to the Jessa AF Knowledge Questionnaire support the qualitative data, as they showed that patients in program B acquired increased knowledge about AF at follow-up compared with baseline. No significant differences were found in the number of electrocardiography recordings between the 2 groups.

**Conclusions:**

Patients with AF and their spouses were positive about the TR program and they found the TR program useful, especially because it created an increased sense of security, knowledge about mastering their symptoms, and a community of practice linking patients with AF and their spouses and health care personnel. To assess all the benefits of the Future Patient–TR Program for patients with AF, it needs to be tested in a comprehensive randomized controlled trial.

**Trial Registration:**

ClinicalTrials.gov NCT04493437; https://clinicaltrials.gov/ct2/show/NCT04493437.

## Introduction

### Background

Atrial fibrillation (AF) is the most common cardiac arrhythmia, occurring in 3% of the adult population worldwide and is predicted to be more than double in prevalence over the next 20 years [1]. The increase in AF prevalence can be attributed to aging of the population, better screening for silent AF, and to an increase in conditions predisposing individuals to AF, such as obesity, hypertension, diabetes, obstructive sleep apnea, and physical inactivity [2]. AF is a chronic disease and a major cause of cardiovascular morbidity and mortality. If untreated, AF is associated with a five-fold increased risk of stroke, and 20%-30% of all strokes are attributable to arrhythmia [1].

Patients with AF experience a variety of symptoms, such as palpitations, fatigue, dyspnea, chest pain, sleeping difficulties, fear, and anxiety. The severity of symptoms varies from individual to individual. Although up to 40% of patients with AF are asymptomatic, others report severe or disabling symptoms [1]. In addition, patients with AF have significantly lower health-related quality of life (QoL) compared with healthy controls [3], and they experience more anxiety compared with patients with other heart diseases [4]. In turn, anxiety may lead to avoidance behaviors and a sedentary lifestyle. Both anxiety and depression in patients with AF have been linked to impaired QoL [3].

Apart from anticoagulation to prevent strokes, the management of patients with AF includes risk factor modification and reduction of symptoms and measures to improve their QoL [5,6]. Hence, the evaluation of QoL is an important part of disease management in patients with AF.

Tailored patient education is recommended as an important aspect of AF care. Current guidelines for patients with AF emphasize measures enabling these patients to become more active participants in the management of their own disease [1,3]. In addition, patient knowledge about AF, risk factors, treatment, and self-management strategies are key factors enabling patients to feel more informed, involved, and empowered in relation to self-care and disease management. Patient acceptance of their AF treatment plan will affect their coping abilities and increase their adherence to the recommended therapy. Education of patients and their spouses is therefore essential not only for their understanding of the disease but also for empowering patients to participate in shared decision making and for encouraging their self-management role in relation to recurrent symptomatic AF [7].

Patients with AF report that they do not receive sufficient education or assistance from health care professionals regarding how to live with their AF [7,8]. Moreover, various studies have demonstrated that patients with AF often have poor knowledge of the arrhythmia, how it can be treated, and how to self-manage their disease [7,9,10]. Conventional cardiac rehabilitation has shown benefits in other chronic cardiovascular conditions, demonstrating significant reductions in cardiovascular mortality and rehospitalizations as well as improvements in health-related QoL [11]. In 2019, Denmark launched the first national strategy for the rehabilitation of patients with AF. However, the Danish health care system does not yet offer rehabilitation programs for patients with AF [12]. Standardized care for patients with AF in the Danish health care system consists of visits to doctors and nurses in outpatient AF clinics. During these visits, the patients received advice and education on living with AF and anticoagulation therapy. After the patients with AF have completed their orientation at the AF clinic, they can contact their own general practitioner if needed. With this gap in rehabilitation offerings, there is an urgent need to develop and test new rehabilitation programs for patients with AF. To address this shortcoming, telerehabilitation (TR) may be a new innovative strategy that may be useful in the COVID-19 context.

TR is defined as rehabilitation using information and communication technologies for delivery of rehabilitation activities [13]. Reviews describing TR in cardiac patients highlight the findings that TR has been shown to be as effective as conventional rehabilitation [14,15]. A review of the literature showed no studies of TR programs for AF patients that included monitoring parameters such as electrocardiography (ECG), steps, sleep, blood pressure, pulse, and weight. Moreover, no studies were found on TR programs that included patient education for patients with AF.

### Objective

Between 2016 and 2019, our research group developed and tested the Future Patient (FP) program for patients with heart failure (HF) in a participatory design process [16-18]. The outcome of this process was the development of a TR program using a web-based digital toolbox and communication platform the *HeartPortal*, reported in Joensson et al [19]. The design of HeartPortal is based on a self-determination theory, which conceptualizes how patients experience feelings of autonomy, competency, and relatedness in relation to their disease management. A high level of self-determination is essential for sustained patient motivation [20].

The aim of this pilot study is to evaluate and compare the feasibility of the 2 TR programs for patients with AF.

## Methods

### FP-TR Program

Two FP programs for patients with AF (FP-AF), A and B, have been developed based on a review of the literature, clinical guidelines [1,12], and a participatory design process [16,17,21]. The pilot phase was conducted between December 2019 and March 2020. The AF clinic at Viborg and Skive Regional Hospital in Denmark and the health care centers in Viborg and Skive Municipalities participated in the pilot study. On the basis of the participatory design process, 2 TR programs, A and B, were developed and are described in the section below.

### Presentation of Programs for TR of AF

**Figure 1:**
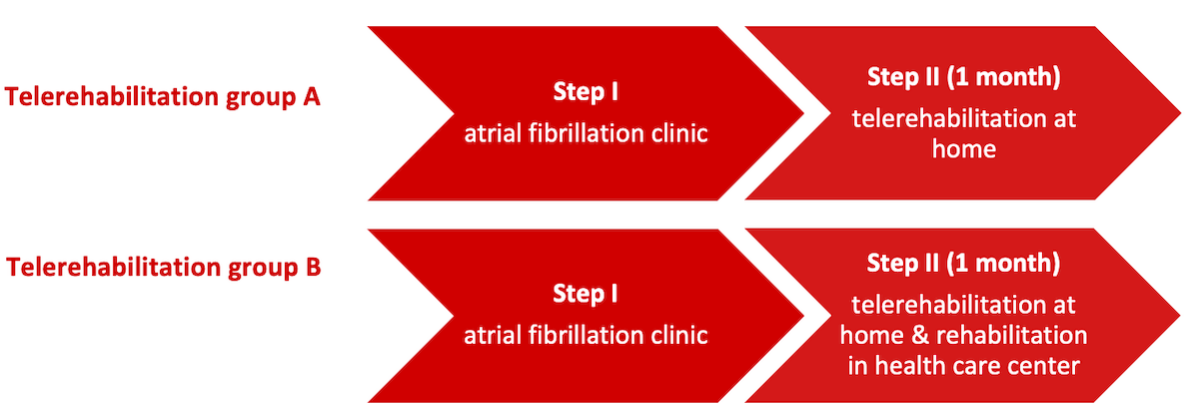
Telerehabilitation in two phases.

The pilot study consisted of testing programs A and B in two phases: (1) initial treatment at the AF clinic and (2) TR at home. The elements of the 2 TR programs are presented in Textbox 1. Programs A and B differed primarily in relation to patient education, as patients in program B participated in rehabilitation at the health care center during phase 2 in the form of four closed sessions focusing on patient education, whereas patients in program A received brief individual instruction by a nurse. Each educational session lasted 2 hours. The topics covered during these sessions included knowledge of AF, AF medication, AF attacks, mental health, lifestyle changes, and body awareness. These topics were chosen based on the recommendations from national guidelines [7,12] regarding patient education aimed at AF disease management. The two phases and their contexts are illustrated in Figure 1.

**Figure 2:**
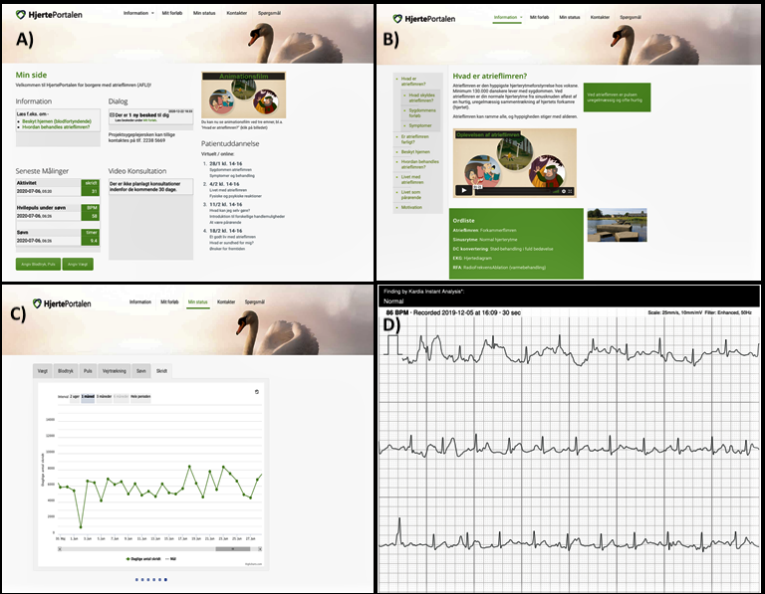
Screen captures of selected information pages from the HeartPortal (Danish: Hjerteportalen.dk). (A) My page (Danish: Min side) with the latest measurement and access to dialogue and video consultation; (B) information module with information about what is AF? (Danish: Hvad er atrieflimren?); (C) overview of steps taken; and (D) overview of heart rhythm from the electrocardiography monitor.

Both groups received a blood pressure device (iHealth Neo), weight scale (iHealth Lina), sleep sensor (Emfit QS), step counter (Fitbit Inspire or Charge 3), an iPad (Apple iPad Air 2), and an ECG monitor (AliveCor KardiaMobile). Furthermore, the 2 groups obtained access to the HeartPortal web portal, which is a digital toolbox that functions as an interactive learning module. Screenshots of selected information sites of the HeartPortal are shown in Figure 2. The module consists of an interactive information site for patient education, a communication platform enabling patients to communicate directly with health care professionals through chat or video consultations with health care professionals, a self-tracking module with visualization of measured data, and questionnaires. These devices were chosen based on the FP-TR program for patients with HF [22].

**Presentation of telerehabilitation programs A and B.**

**Telerehabilitation Program A**

- Content of the program and education of patients and spouses
  At enrollment, the project nurse orients the patients and spouses briefly on the following topics knowledge of atrial fibrillation (AF), AF medication, AF attacks, mental health, lifestyle changes, and body awareness. The project nurse encourages the patients and spouses to study the information module at the HeartPortal, where they can read more about the topics.
- Technologies
  Blood pressure device
  Weight scale
  Sleep sensor
  Step counter
  iPad
  Electrocardiography monitor
- Communication platform
  Dialogue and video module at the HeartPortal among patients, the AF clinic at the hospital, and health care centers.
  Patients, spouses, health care professionals from the AF clinic at the hospital and health care professionals from the health care centers had access to the HeartPortal.
- Overview of monitored data and rehabilitation plan
  Graphic module with overview of measured data at the HeartPortal.
  Patient can design their own rehabilitation plan.

**Telerehabilitation Program B**

- Content of the program and education of patients and spouses
  Patients and spouses were offered to participate in rehabilitation at the health care center in the form of four closed sessions focusing on patient education. The topics during these sessions included knowledge of AF, AF medication, AF attacks, mental health, lifestyle changes, and body awareness. Each session lasted 2 hours, and the teaching was carried out by a nurse from the AF clinic at the hospital and by physiotherapists and a psychologist from the health care center. The patients and spouses were encouraged to study the information module at the HeartPortal, where they can read more about the topics.
- Technologies
  Blood pressure device
  Weight scale
  Sleep sensor
  Step counter
  iPad
  Electrocardiography monitor
- Communication platform
  Dialogue and video module at the HeartPortal among patients, the AF clinic at the hospital, and health care centers.
  Patients, spouses, health care professionals from the AF clinic at the hospital, and health care professionals from the health care centers had access to the HeartPortal.
- Overview of monitored data and rehabilitation plan
  Graphic module with overview of measured data at the HeartPortal.
  Patient can design their own rehabilitation plan.

### Outcomes

The following primary and secondary outcomes have been defined in the pilot study:

1. Primary outcome:
  - Usability of technologies and content of the TR program seen from patients’ perspectives.
2. Secondary outcomes:
  - Patients’ knowledge of AF at baseline and at the end of the study.
  - Development of clinical data over 4 weeks.
  - Patients’ and spouses’ expectations and experiences of participating in the TR program.

### Ethical Considerations

This pilot study was approved by the North Denmark Region Committee on Health Research Ethics (N-20190059) and is listed on ClinicalTrials.gov (NCT04493437). The study was conducted in accordance with the Declaration of Helsinki. All participants signed an informed consent form before enrollment in the study.

### Participants and Recruitment

The target group of the FP-AF pilot study included patients diagnosed with AF. The patients were recruited from AF clinics at the Viborg and Skive Regional Hospital, Denmark. Patients were eligible for the study if they were diagnosed with AF, were adults above 18 years of age, were living in Viborg or Skive Municipality, were living at home and capable of caring for themselves, and had basic computer skills or a spouse with basic computer skills. Patients were excluded if they were pregnant, lacked the ability to cooperate, or had insufficient basic Danish language skills.

**Figure 3:**
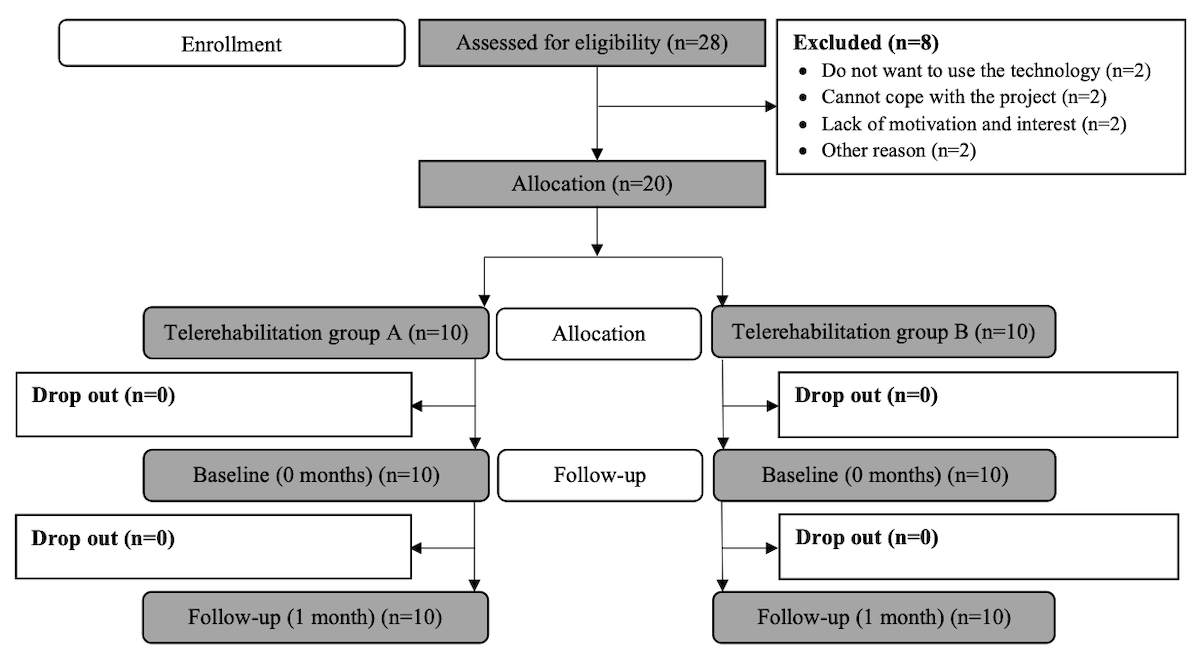
Consolidated Standards of Reporting Trials (CONSORT) diagram of the pilot study.

In total, 20 patients with AF were included in the FP-AF pilot study, of which the first 10 patients were allocated to participate in program A, and the next 10 patients were allocated to participate in program B. The allocation and follow-up of the patients are shown in the CONSORT (Consolidated Standards of Reporting Trials) diagram illustrated in Figure 3.

### Data Collection

Sociodemographic and clinical data were acquired from the patients’ medical journal or through self-reporting.

### Interviews

Semistructured qualitative interviews, inspired by Brinkmann and Kvale [23], were conducted at the end of the study with patients and spouses in programs A and B at the patients’ homes by the first (BD) and second author (JDG). The aim of the interviews was to collect data on how patients viewed the usability of the technologies, the content of the TR programs, and the experiences of the patients and spouses participating in the FP-AF. All 10 patients in program A but only 9 patients in program B participated in the interviews, as did 11 of their spouses. Each interview lasted 30-40 minutes and was tape-recorded. The interviews were transcribed and documented in word files.

### Questionnaires

All questionnaire data were collected using the Research Electronic Data Capture (Vanderbilt). To evaluate the usability of HeartPortal, a 5-point Likert scale questionnaire was used, covering both the usability and design aspects of HeartPortal [24]. The questionnaire was validated in a previous study [19]. All patients in programs A and B completed the questionnaire.

Data regarding patients’ AF knowledge were collected using the Jessa AF Knowledge Questionnaire (JAKQ) [10,25]. These questionnaires were web-based and answered by patients in both groups, both at baseline and at the end of the study. The answers from the JAKQ were scored on a 4-point Likert scale. The questionnaire responses were extracted from REDCap (Research Electronic Data Capture).

### Clinical Measures

The self-monitored data from the devices that the patients had used at home were acquired from Fitbit, iHealth, and Emfit using an application programming interface, whereas the self-monitored ECG data were acquired from KardiaPro. After an ECG measurement, the Alivecor Kardia software displayed feedback to the patient showing whether the measurement had been classified as *Normal ECG,* *Possible AF,* or *Unclassified*.

### Statistical Analysis

Before the statistical analyses, data were examined for normality of distribution using the Shapiro-Wilk test.

The sociodemographic and clinical data at baseline and the number of ECG measurements were compared between the 2 groups using an independent samples *t* test (two-tailed) for normally distributed variables and a Mann-Whitney test for nonnormally distributed variables.

The JAKQ answers were recalculated into a percentage score, such that a higher percentage indicated higher AF knowledge. The questionnaire data from JAKQ were compared between the 2 groups at baseline and at follow-up using a Mann-Whitney test, and within the 2 groups from baseline to follow-up using a Wilcoxon signed-rank test.

Data preprocessing was performed using MATLAB version R2019a, and statistical analyses were performed using IBM SPSS Statistics version 26. A significance level of α=.05 was adopted for all analyses.

### Analysis of Qualitative Data

The transcribed interviews were coded and analyzed using NVivo 12.0, inspired by Brinkmann and Kvale [23]. The findings are reported in themes, subthemes, and citations.

## Results

### Patient Characteristics

The sociodemographic and clinical patient characteristics of both groups at baseline are depicted in Table 1 either as the number of patients or as the means and SDs for the different parameters. The test statistics from the comparison between the characteristics of the 2 groups are shown in Table 1. At baseline, there were no significant differences among patients in programs A and B, except for the resting pulse, which was significantly higher in patients in program A (*P*=.01; Table 1).

**Table 1:** Sociodemographic and clinical patient characteristics at baseline for the patients in program A and B (N=20).

Variable			Program A (N=10)		Program B (N=10)		*P* value	
**Age (years) by gender, mean (SD); n**								
	Men	68.4 (3.29); n=5		70.88 (5.69); n=8		.40		
	Women	74 (4.3); n=5		66.50 (7.78); n=2		.14		
	Men and women	71.2 (4.66); n=10		70 (5.94); n=10		.62		
**Clinical parameters, mean (SD)**								
	Height (cm)	174.40 (7.66)		180 (6.82)		.10		
	Weight (kg)	86.80 (15.58)		90.2 (20.25)		.68		
	Systolic blood pressure (mm Hg)	134.70 (16.39)		143.3 (20.51)		.33		
	Diastolic blood pressure (mm Hg)	83 (14.95)		81.4 (16.47)		.82		
	Resting pulse (beats/min)	84 (21.88)		61.4 (14.37)		.01^a^		
	Ejection fraction (%)	58.50 (4.74)		58.5 (3.38)		.63		
	CHA_2_DS_2-_VASc-score^b^	2.50 (1.08)		1.9 (1.29)		.34		
	HADS-BLED-score^c^	1.80 (0.63)		1.2 (0.79)		.10		
	EHRA^d^-score	2.10 (0.74)		2.2 (0.63)		.77		
	Former DC^e^ (quantity)	0.5 (1.08)		1.3 (1.77)		.12		
	Former RFA^f^ (quantity)	0.80 (1.48)		0.4 (0.7)		.82		
	P-creatinine (µmol/L)	69.40 (17.13)		92.1 (33.45)		.11		
	B-hemoglobin (mmol/L)	8.88 (0.81)		9.2 (0.75)		.68		
	TSH^g^ (×10^−3^ IU/L)	1.70 (0.7)		1.72 (1.23)		.60		
	Years with AF^h^	4 (5.831)		5.8 (6.27)		.18		
**Primary diagnoses, n (%)**								.99
	Paroxysmal AF	8 (80)		8 (80)				
	Persistent AF	1 (10)		1 (10)				
	Permanent AF	1 (10)		1 (10)				
**Secondary diagnosis, n (%)**								.79
	Hypertension	3 (30)		5 (50)				
	Diabetes mellitus	0 (0)		0 (0)				
	Former stroke or TIA^i^ peripheral embolism	2 (20)		0 (0)				
	Ischemic heart disease	0 (0)		0 (0)				
	Claudication	0 (0)		0 (0)				
**Civil status, n (%)**								.07
	Single or living alone	2 (20)		0 (0)				
	Married or living with a partner	8 (80)		10 (100)				
**Education, n (%)**								.59
	Primary school	0 (0)		1 (10)				
	Unskilled	0 (0)		0 (0)				
	Skilled worker	4 (40)		4 (40)				
	High school	0 (0)		0 (0)				
	Bachelor’s degree	5 (50)		4 (40)				
	Master’s degree	1 (10)		1 (10)				
	At least PhD	0 (0)		0 (0)				
**Work status, n (%)**								.65
	Unemployed	0 (0)		0 (0)				
	Sick leave	0 (0)		0 (0)				
	Works under 20 hours/week	0 (0)		0 (0)				
	Works 20-36 hours/week	1 (10)		1 (10)				
	Works full-time 37 hours/week	1 (10)		2 (20)				
	Retired	8 (80)		7 (70)				

^a^Indicates significant test statistics (*P*=.05).

^b^CHA_2_DS_2-_VASc-score.

^c^HADS-BLED-score.

^d^EHRA: European Heart Rhythm Association.

^e^DC: direct current cardioversion.

^f^RFA: radiofrequency ablation.

^g^TSH: thyroid-stimulating hormone.

^h^AF: atrial fibrillation.

^i^TIA: transient ischemic attack.

### Patients’ Experiences

Patients’ experiences were evaluated based on qualitative data from their interviews. The interview data were categorized into the following themes: user-friendliness of the technologies, usage of the HeartPortal and preferences in acquiring information (Textbox 2).

In Tables 2 and 3, most of the patients in programs A and B responded that HeartPortal has a high degree of usability in relation to navigation, easy information, and logical structure.

Tables 4 and 5 demonstrate that patients in program B highlight the importance of education at the health care center for them and their spouses. They found education to be useful and relevant.

**Findings from interviews with patients with atrial fibrillation and their spouses.**

**Patients’ Perspectives in Themes**

The portal is a useful digital tool when you need to learn to live with atrial fibrillation.

- Usefulness of the HeartPortal.dk
  Information is easy to understand
  Animation video communicating knowledge about life with atrial fibrillation (AF) in a simple way
  Data give me an overview of the progress of my rehabilitation
  The portal is a good tool for AF rehabilitation

When I learn about my disease and symptoms, I feel secure living with atrial fibrillation.

- Feeling more secure living with AF
  Learning about my disease creates a sense of security

Meeting other patients and spouses gives me a feeling of not being alone; my wife and I learn from the other participants.

- Community of practice between patients living with AF
  Teaching at the health care center creates a feeling of cohesion
  Mutual interest and learning among patients and spouses

I feel secure when I can see how my heart is beating.

- Measuring heart rhythm makes good sense
  Feeling of security
  Need more knowledge about how to read the electrocardiography

**Spouses Perspectives in Themes**

There is useful information in the HeartPortal on how to live with atrial fibrillation as a patient and for me as a spouse. I like that it is also communicated in animation videos.

- Increased knowledge about AF and how to support spouse living with AF
  HeartPortal is a useful toolbox
  Feeling of security

During the education at the health care center, I met other spouses and we formed relationships and felt confident sharing experiences.

- Community of practice between spouses
  Knowledge sharing with other spouses is useful
  Exchange of ideas on how to support spouse living with AF

**Table 2:** Patients’ responses to usability of the HeartPortal.

Variable: usability of the HeartPortal	Strongly agree, n (%)	Agree, n (%)	Neutral, n (%)	Disagree, n (%)	Strongly disagree, n (%)
It is easy to navigate on the HeartPortal	8 (42.11)	6 (31.58)	4 (21.05)	1 (5.26)	0 (0)
The information is understandable	13 (68.42)	6 (31.58)	0 (0)	0 (0)	0 (0)
I gain new knowledge about AF^a^ from the videos	6 (31.58)	4 (21.05)	4 (21.05)	5 (26.32)	0 (0)
The HeartPortal is logically structured	12 (63.16)	5 (26.32)	0 (0)	2 (10.52)	0 (0)
The buttons have a suitable size	12 (63.16)	7 (36.84)	0 (0)	0 (0)	0 (0)

^a^AF: atrial fibrillation.

**Table 3:** Patients’ responses to the design of the HeartPortal.

Variable: design of the HeartPortal	Excellent, n (%)	Very good, n (%)	Good, n (%)	Bad, n (%)	Very bad, n (%)
Text size	13 (68.42)	4 (21.06)	2 (10.52)	0 (0)	0 (0)
Amount of text	7 (36.84)	7 (36.84)	5 (26.32)	0 (0)	0 (0)
Color scheme	12 (63.16)	4 (21.05)	3 (15.79)	0 (0)	0 (0)
Length of the videos	13 (68.42)	1 (5.26)	4 (21.05)	1 (5.26)	0 (0)
Structure of the HeartPortal	12 (63.16)	2 (10.52)	5 (26.32)	0 (0)	0 (0)

**Table 4:** Response of patients in program B to patient education at the health care center^a^.

Variable	Strongly agree, n (%)	Agree, n (%)	Neutral, n (%)	Disagree, n (%)	Strongly disagree, n (%)
I have gained more knowledge on living with AF^b^	5 (55.56)	4 (44.44)	0 (0)	0 (0)	0 (0)
The education has helped me feel more comfortable living with AF	6 (66.67)	3 (33.33)	0 (0)	0 (0)	0 (0)
The education complements the HeartPortal	5 (55.56)	4 (44.44)	0 (0)	0 (0)	0 (0)
It has been important for me to have my spouses with me at the health care center	4 (44.44)	1 (11.11)	3 (33.33)	0 (0)	1 (11.11)
The topics have been relevant for me	7 (77.78)	1 (11.11)	1 (11.11)	0 (0)	0 (0)

^a^One person did not participate in the interview.

^b^AF: atrial fibrillation.

**Table 5:** Response of patients in program B to patient education at the health care center^a^.

Variable	Excellent, n (%)	Very good, n (%)	Good, n (%)	Bad, n (%)	Very bad, n (%)
How have you experienced the education at the health care center?	7 (77.78)	1 (11.11)	1 (11.11)	0 (0)	0 (0)
What do you think about the form of the education?	5 (55.56)	3 (33.33)	1 (11.11)	0 (0)	0 (0)

^a^One person did not participate in the interview.

### AF Knowledge

A Wilcoxon signed-rank test was used to test whether a statistically significant difference was present when comparing the median scores (%) and IQR of the JAKQ scores at baseline and follow-up individually in the 2 groups, and on this basis. Patients in program B showed statistically significantly higher AF knowledge (*P*=.02) at follow-up (median 86.06, IQR 22.36) compared with baseline (median 69.23, IQR 21.88), whereas no difference was found for patients in program A (baseline: median 78.13, IQR 31.25; follow-up: median 81.25, IQR 25; *P*=.13).

### Clinical Measures

During the intervention, both groups performed self-monitoring of the clinical measurements. The mean and SD of these measurements were evaluated for each week of the intervention for both patients in program A and those in program B. The results are shown in Table 6.

**Table 6:** Clinical measures as monitored by the patients for program A and program B.

Variable			Week 1, mean (SD)		Week 2, mean (SD)		Week 3, mean (SD)		Week 4, mean (SD)
**Program A**									
	Systolic blood pressure (mm Hg)	138.5 (11.15)		142.85 (19.62)		137.28 (11.24)		134.67 (13.03)	
	Diastolic blood pressure (mm Hg)	82.8 (11.13)		84.95 (8.89)		81.11 (10.31)		82.28 (10.85)	
	Pulse (beats per min)	76.3 (14.18)		73.95 (17.11)		68.61 (13.37)		71.78 (14.27)	
	Weight (kg)	88.38 (15.69)		90.6 (16.39)		89.25 (16.95)		90.06 (18.32)	
	Daily steps (n)	7150.5 (5370.17)		8443.4 (6069.01)		5993.55 (4172.97)		7236.50 (5110.75)	
	Distance (km)	5.33 (4.01)		6.29 (4.51)		4.45 (3.08)		5.39 (3.82)	
	Pulse during sleep (beats per min)	63.86 (8.59)		63.73 (8.6)		63.75 (8.64)		63.9 (7.76)	
	Respiration during sleep (breaths per min)	15.41 (1.9)		15.12 (1.98)		15.02 (2.1)		15.05 (1.85)	
	Sleep score (%)	70.5 (12.41)		82.25 (16.13)		84.05 (15.3)		78.59 (20.62)	
	Sleep time (hours)	7.09 (1.6)		7.56 (1.43)		7.68 (1.21)		8.02 (1.75)	
**Program B**									
	Systolic or diastolic blood pressure (mm Hg)	139.25 (18.04)/82.3 (12.93)		135.22 (14.69)/79.94 (10.13)		129.28 (12.92)/78.11 (6.16)		127.33 (16.3)/77.72 (10.99)	
	Pulse (beats per min)	66.35 (11.08)		66.39 (10.72)		66.56 (11.9)		64.78 (7.6)	
	Weight (kg)	88.42 (20.44)		90.15 (20.04)		89.38 (21.27)		90.68 (22.69)	
	Steps	8638.35 (4783.43)		8683.00 (5003.07)		7585.85 (4959.23)		7565.25 (3982.92)	
	Distance (km)	6.41 (3.55)		6.44 (3.76)		5.64 (3.69)		5.64 (2.98)	
	Pulse during sleep (beats per min)	57.98 (4.58)		58.61 (4.91)		59.17 (4.55)		60.74 (4.66)	
	Respiration during sleep (breaths per min)	14.44 (1.27)		14.57 (1.3)		14.24 (1.32)		14.29 (1.29)	
	Sleep score (%)	75.16 (18.81)		83.52 (8.87)		78.81 (17.27)		78.68 (13.16)	
	Sleep time (hours)	7.59 (1.5)		7.74 (0.68)		7.83 (0.8)		7.76 (0.9)	

The median number of ECG recordings and classifications and the IQR are shown in Table 7. In addition, the difference between the 2 groups was statistically analyzed using the Mann-Whitney test, the results of which are also shown in Table 7. The results showed that there were no statistical differences in the number of ECG recordings or classifications between the patients in programs A and B.

**Table 7:** Median number of electrocardiography recordings for groups A and B and IQR and results of the Mann-Whitney tests.

Variable	Program A, median (IQR)	Program B, median (IQR)	*P* value
Total number of recordings	19 (20)	19 (19)	.85
Normal ECG^a^	7 (16)	7.5 (14)	.70
Possible AF^b^	10 (13)	5.5 (23)	.82
Unclassified	3 (6)	4 (6)	.94

^a^ECG: electrocardiography.

^b^AF: atrial fibrillation.

## Discussion

### Principal Findings

The aim of this pilot study was to evaluate and compare the feasibility of the 2 TR programs for patients with AF. The sociodemographic and clinical patient characteristics showed that the 2 groups in the study were comparable at baseline (Table 1). Through interviews, patients articulated the following themes (Textbox 2) about their participation in a TR program: the HeartPortal is a useful tool, increased feeling of security while living with AF, being part of a community of practice living with AF, and measuring one’s heart rhythm makes good sense in AF disease management. The findings from the FP-AF study are in line with the findings from a qualitative study by Dinesen et al [22] on TR in patients with HF. These patients stated that TR technologies and access to their own data provided a relevant overview for the patients in relation to their rehabilitation processes. Furthermore, the patients stated that TR encouraged them to carry out activities on their own, and that they felt more at ease in performing their rehabilitation activities outside the hospital and the health care centers [26]. Other data from the same study comparing psychological aspects across conventional rehabilitation and rehabilitation were reported by Spindler et al [27], where conventional rehabilitation therapy versus TR in patients with HF was compared [27]. They showed that patients in both groups were equally motivated for lifestyle changes and self-care, and that they experienced similar levels of psychological distress and QoL. Spindler et al [27] concluded that based on psychological measures, TR may be a feasible alternative to conventional rehabilitation. The previous study can help us take the next step in testing FP-AF [27].

Through interviews, the spouses of patients with AF expressed that they had gained increased knowledge about AF and on how to support their spouses to cope with their AF in everyday life. The spouses (Textbox 2) also expressed that they felt more like part of a community of practice with the other spouses participating in education at the health care center. The qualitative study by Dinesen et al [22] on TR in patients with HF also explored how their spouses participated in a TR program. They found that the spouses had an increased sense of security, they took too much responsibility on behalf of their partner, and that they tended to push their partner too hard at times. As such, Dinesen et al [22] suggested that it is important to identify the most effective ways of involving spouses when designing a new cardiac TR program. The spouses certainly need to acquire sufficient knowledge and education about the disease of their partner, and they need to find the best way to help their partner prevent worsening of their symptoms [26].

On the basis of questionnaire responses, the patients reported that they found the HeartPortal easy to navigate, that the information provided was understandable, that the animation videos helped them gain new knowledge about AF, that the HeartPortal was logically structured, and that the design of the HeartPortal was assessed as very good or excellent (Tables 2 and 3). In addition, patients in program B expressed the view that the education at the health care center helped them gain more knowledge about living with AF, and they valued having their spouses participating in the sessions at the health care center (Tables 4 and 5). These results are supported by findings based on the AF knowledge questionnaire, JAKQ, showing that patients in program B acquired increased knowledge about AF at follow-up compared with baseline. These results are comparable with findings from another study using the JAKQ to evaluate the effectiveness and usability of an online tailored education platform to inform patients with AF undergoing direct current cardioversion or pulmonary vein isolation [25]. This study found that AF-related knowledge in patients who received online education was significantly better after 6 weeks, whereas no significant differences over time were found in the group that received online standard care. A review of digitalized patient education for patients with HF, coronary artery disease, and AF concluded that digital education increased QoL, increased knowledge, and decreased depression and anxiety [28]. In addition, the review by Oudkerk Pool et al [28] highlighted that patients are satisfied with digital platforms. However, the review only included 1 pilot study with 100 patients with AF; therefore, there is an urgent need for more knowledge of digitalized patient education for patients with AF in TR programs.

Kayser et al [29] stressed that in a matrix framework for designing digital technologies and services for patients with chronic conditions, they needed to view the patients’ role more broadly, in terms of engagement, empowerment, and emancipation. In programs A and B of the FP-AF, we attempted to design the HeartPortal, remote monitoring, and educational modules to be interactive and motivating. In addition, we educate health care professionals to help facilitate empowerment and emancipation. These issues will be addressed in a future larger study of the FP-AF program.

Clinical data are shown in Table 6 for groups A and B at both the baseline and follow-up stages. The same number of ECG recordings was carried out for patients in programs A and B, and there was an equal number of normal ECGs in the 2 groups (Table 7). In program A, a median of 10 cases of possible AF were identified, and in program B, the median of possible cases of AF was 5.5. However, no significant differences were found in the ECG recordings of the 2 groups. When designing a TR program, the burden of tracking arises as a question. For patients with AF, we questioned whether patients would benefit from measuring their ECG at home and whether this measurement activity would be a burden for the patient. In our qualitative interviews with patients with AF, they expressed the view that they felt secure measuring their own ECG; however, they needed more knowledge about how to read the ECGs. We identified a new European TeleCheck-AF mobile health study that began as a response to the COVID-19 pandemic [30]. The focus of the TeleCheck-AF study was on remote AF and risk factor management through teleconsultation. In that study, patients with AF were asked to measure their heart rhythm and heart rate for 7 days before a scheduled teleconsultation with a doctor at the hospital. The TeleCheck-AF study is ongoing in several European countries, but the results are not yet available. We have not identified other studies with a focus on TR for patients with AF using components such as remote monitoring, a web-based interactive platform, or education at a health care center.

On the basis of our experiences with using participatory design for TR programs, we also used this approach as an overall method for developing the FP program and the HeartPortal in collaboration among patients, spouses, and health care professionals [19,22]. Program B (Textbox 2) of the TR program was chosen by the patients as the best program in terms of content and structure, as they value having education at the health care center with their spouses as a part of the TR program.

### Limitations

One limitation of this study is that the pilot phase lasted for only 1 month. Program B of the FP-AF will have to be tested for a longer period by both patients with AF and spouses, and further, in a randomized controlled trial to generate sufficient evidence about the effects of the program. The pilot study has been tested only on Danish patients, which is a limitation, as the results may not readily be generalized to other cultural contexts at this stage.

### Conclusions

Overall, patients with AF and their spouses were positive about participating in a TR program consisting of remote monitoring, an interactive web-based HeartPortal, and education at a local health care center. Patients with AF and their spouses found the TR program useful, especially because it created an increased sense of security, enhanced their knowledge about mastering their symptoms, and a feeling of belonging to a community of practice linking patients with AF and their spouses and health care personnel. To assess the full benefits of FP-AF, this TR program needs to be tested in a comprehensive randomized controlled trial.

## Abbreviations

AF: atrial fibrillation
CONSORT: Consolidated Standards of Reporting Trials
ECG: electrocardiography
FP: future patient
FP-AF: FP telerehabilitation program for patients with AF
HF: heart failure
JAKQ: Jessa AF Knowledge Questionnaire
QoL: quality of life
REDCap: Research Electronic Data Capture
TR: telerehabilitation
